# COVID-19 epidemic investigation study of a follow-up cohort of patients with diabetic kidney disease

**DOI:** 10.3389/fcimb.2024.1388260

**Published:** 2024-08-20

**Authors:** Qian Wang, Zheyi Dong, Weiguang Zhang, Ying Zheng, Qiang Lyu, Ruimin Zhang, Hui Huang, Fang Liu, Yong Wang, Li Zhang, Xueying Cao, Jie Wu, Jianhui Zhou, Guangyan Cai, Xiangmei Chen

**Affiliations:** Department of Nephrology, First Medical Center of Chinese PLA General Hospital, Nephrology Institute of the Chinese People’s Liberation Army, State Key Laboratory of Kidney Diseases, National Clinical Research Center for Kidney Diseases, Beijing Key Laboratory of Kidney Disease Research, Beijing, China

**Keywords:** coronavirus disease, diabetic kidney disease, diabetic nephropathy, infection rate, vaccination

## Abstract

**Introduction:**

The impact of coronavirus disease 2019 (COVID-19) on diabetic kidney disease (DKD) patients in China is not fully understood. This study aimed to investigate infection status in a DKD cohort post-renal biopsy and analyze vaccination and infection rates, as well as symptom severity, across various renal pathologies in DKD patients.

**Methods:**

This epidemiological survey, centered on COVID-19, employed a Chinese DKD and renal puncture follow-up cohort. A customized questionnaire enabled standardized data gathering. It collected data on clinical characteristics, vaccination and infection statuses, and diverse pathological types. The study analyzed the relationship between vaccination and infection statuses across various pathological types, evaluating characteristics and treatment outcomes in patients with infections.

**Results:**

In total, 437 patients with DKD from 26 Chinese provinces were followed up for a median of 44.6 ± 20 months. COVID-19 infection, vaccination, and novel coronavirus pneumonia (NCP) rates were 73.68%, 59.3%, and 6.63%, respectively. Ten patients with NCP had severe pneumonia or died of COVID-19. Renal pathology revealed that 167 (38.22%) patients had diabetic nephropathy (DN), 171 (39.13%) had non-diabetic renal disease (NDRD), and 99 had DN and NDRD (22.65%). The DN group had the lowest vaccination (54.5%), highest all-cause mortality (3.6%), and highest endpoint rates (34.10%). Compared to patients who were not vaccinated pre-infection (117 cases), vaccinated patients (198 cases) had reduced NCP (6.6% vs. 13.7%), severity (1.0% vs. 3.4%), and endpoint (9.10% vs. 31.60%) rates.

**Conclusion:**

Vaccination can prevent infection and diminish COVID-19 severity in patients with DKD; therefore, increasing vaccination rates is particularly important.

**Clinical Trial registration:**

ClinicalTrails.gov, NCT05888909.

## Introduction

The novel severe acute respiratory syndrome coronavirus 2 (SARS-CoV-2) virus began spreading in December 2019, initiating the coronavirus disease 2019 (COVID-19) pandemic that has impacted many aspects of human society (Chaw et al., 2020; Wiersinga et al., 2020). In 2022, COVID-19 was either the primary cause or a contributing factor in 0.24 million deaths in the United States (Ahmad et al., 2023). As of July 7, 2024, the World Health Organization (WHO) has reported 775.67 million confirmed cases of COVID-19, including 7.05 million deaths (W.H.O, 2023). In China, from January 3, 2020, to July 7, 2024, there were 99.4 million confirmed cases of COVID-19, resulting in 0.12 million deaths (W.H.O, 2023).

Patients with diabetes are at a high risk of infection with SARS-CoV-2. Older adult patients with comorbidities, including hypertension, diabetes, asthma, chronic obstructive pulmonary disorder, and chronic kidney disease, are at increased risk of developing severe COVID-19 and adverse clinical outcomes (Akhtar et al., 2021; Salinas-Aguirre et al., 2022). Age (Yang et al., 2020) and underlying diseases (including hypertension and diabetes) (Li et al., 2020) are the most important risk factors for COVID-19-related death. Type 2 diabetes mellitus-associated diabetic nephropathy (DN) and severe COVID-19 are known to be closely associated (Mourad et al., 2021), with DN increasing the risk of COVID-19-related death (Hadjadj et al., 2023). Patients with diabetic kidney disease (DKD) have increased rates of COVID-19, intensive care unit admission, and death compared to patients with chronic kidney disease (CKD) alone. Further, lung infection and intubation rates of those with DKD are twice those of patients with CKD (Abdulaziz Al-Muhanna et al., 2022).

In the early stages of the epidemic, SARS-CoV-2 was highly pathogenic, with pneumonia commonly seen in the clinic. Variations in the virus weakened its virulence, particularly after the Omicron viral strain predominated (Fan et al., 2022; Qu et al., 2023). Human infection manifestations currently include cough, fever, and sore throat, with few developing pneumonia. Over 3 years, China’s government has implemented protective public health policies. On January 6, 2023, China’s National Health Commission and the National Administration of Traditional Chinese Medicine released the updated Diagnosis and Treatment Plan for Novel Coronavirus Infection, in line with Class B COVID-19 management and epidemic control measure revisions. This plan renamed the disease from “novel coronavirus pneumonia” (NCP) to “novel coronavirus infection” and incorporated a positive COVID-19 antigen test into the diagnostic criteria (NHC, NATCM, 2023).

Since December 2022, China’s epidemic prevention has shifted from infection prevention to health protection and severe case prevention. Efforts have been made to administer medical treatment, enhance COVID-19 cure rates, and lower mortality rates (NHC, NATCM, 2023). The effect of COVID-19 on DKD patients in China post-policy change is yet to be determined. To understand this, an epidemiological survey was conducted on a follow-up cohort of patients diagnosed with DKD via renal biopsy, covering 26 provinces.

## Materials and methods

### Participants and survey design

This epidemiological study investigated 437 patients with type 2 diabetes who underwent renal biopsy at the First Medical Center of the People’s Liberation Army General Hospital between April 1, 2017, and November 30, 2022. The inclusion criteria were as follows: aged 18 years or older at renal biopsy for both sexes; diagnosis of type 2 diabetes; and diagnosis of renal lesions. Exclusion criteria included the following: incomplete data, unclear medical history, unclear pathological diagnoses, secondary diabetes, or tumors. The survey was conducted from January to March 2023, with a dedicated survey questionnaire designed for the collection of standardized data from the cohort of patients. Questionnaire data were collected both over the telephone and at outpatient follow-up. This study was approved by the Ethics Committee of the Chinese People’s Liberation Army General Hospital (No. S2022-482-01). Written informed consent was obtained from all participants.

Patients with pathological kidney results were categorized into three groups: DN, non-diabetic renal disease (NDRD), and DN combined with NDRD. They were further classified into infected and uninfected subgroups based on SARS-CoV-2 infection, with infected patients subdivided into vaccinated and unvaccinated. The study compared COVID-19 vaccination and infection status across different DKD pathological types and examined the impact of clinical characteristics on infection status.

### Data collection and evaluation

Standardized data collection tables (**Supplementary Material**), tailored for this research, systematically organized and recorded data. Baseline clinical data, COVID-19 infection status, and vaccination status of renal biopsy patients were collected. Each patient’s detailed medical history, encompassing age, sex, residence, examination results, laboratory tests during renal biopsy, and pathology results, was gathered.

The questionnaire contained: follow-up endpoint status, SARS-CoV-2 infection status, vaccination status (dose times), COVID-19 diagnostic method, pneumonia diagnosis, and COVID-19 severity with symptoms and treatment details. COVID-19 severity was determined mainly based on the Diagnosis and Treatment Plan for Novel Coronavirus Infection (Tenth Edition). Based on a comprehensive analysis of epidemiological history, clinical manifestations, laboratory tests, and other factors, a clinical diagnosis and classification (severity) assessment were made. The included patients met the diagnostic criteria for severe and critically severe clinical classifications and were considered to be severely infected (NHC, NATCM, 2023). Symptoms included fever, cough, sore throat, nasal congestion, headache, muscle pain, shortness of breath, difficulty breathing, nausea, chills, vomiting, and diarrhea (shapeless or watery stools). Each symptom could be marked as nonexistent (score 0) or present (score 1–3). COVID-19 severity: mild (score 1), moderate (score 2), severe (score 3) (**Supplementary Material**).

### Diagnosis and outcome assessment

Kidney disease diagnosis relied on renal pathology. Follow-up endpoints included renal replacement therapy or death from any cause. COVID-19 diagnosis encompassed relevant clinical symptoms and one or more positive tests: COVID-19 nucleic acid, antigen, pSARS-CoV-2 isolation/culture, or a convalescent stage COVID-19-specific IgG antibody level quadruple that of the acute stage (NHC, NATCM, 2023). NCP was designated if the requirements of COVID-19 diagnosis were met and characteristic pneumonia manifestations on lung imaging were visible.

### Statistical analyses

Statistical analysis employed IBM SPSS Statistics for Windows, version 25.0. The Kolmogorov-Smirnov test assessed normality in continuous variables. Normally distributed variables are shown as means ± standard deviation, compared via t-test. Non-normally distributed continuous variables are depicted as medians and interquartile ranges and compared using non-parametric tests. Categorical variables are represented as frequencies and percentages and compared with chi-square tests. To compare the overall distribution of composition ratios of multiple groups, R is used × C, which is composed of multiple samples without a sequence-linked-table χ 2 inspection. Statistical significance was established at P < 0.05.

## Results

### General characteristics of patients

In total, 437 patients with DKD from 26 provinces and municipalities in China were followed for an average duration of 44.6 ± 20 months. Throughout this period, 322 patients (73.68%) contracted COVID-19, whereas 115 (26.32%) did not. Additionally, 266 patients (60.9%) received the COVID-19 vaccine. Among those with COVID-19, 29 patients (6.63%) were diagnosed with NCP, and 10 (2.29%) had severe pneumonia or died.

The distribution of patients across geographical regions of China was as follows: northeast (109, 24.9%), north (248, 56.8%), east (26, 5.9%), central (23, 5.3%), northwest (22, 5.0%), and southwest (9, 2.1%); the vaccination rates across regions were 66.1%, 59.3%, 57.7%, 56.5%, 77.3%, and 22.2%, respectively, and COVID-19 detection rates were 78.0%, 72.2%, 69.2%, 73.9%, 72.7%, and 77.8%, respectively. Infection and vaccination rates among regions did not significantly differ.

**Figure 1:**
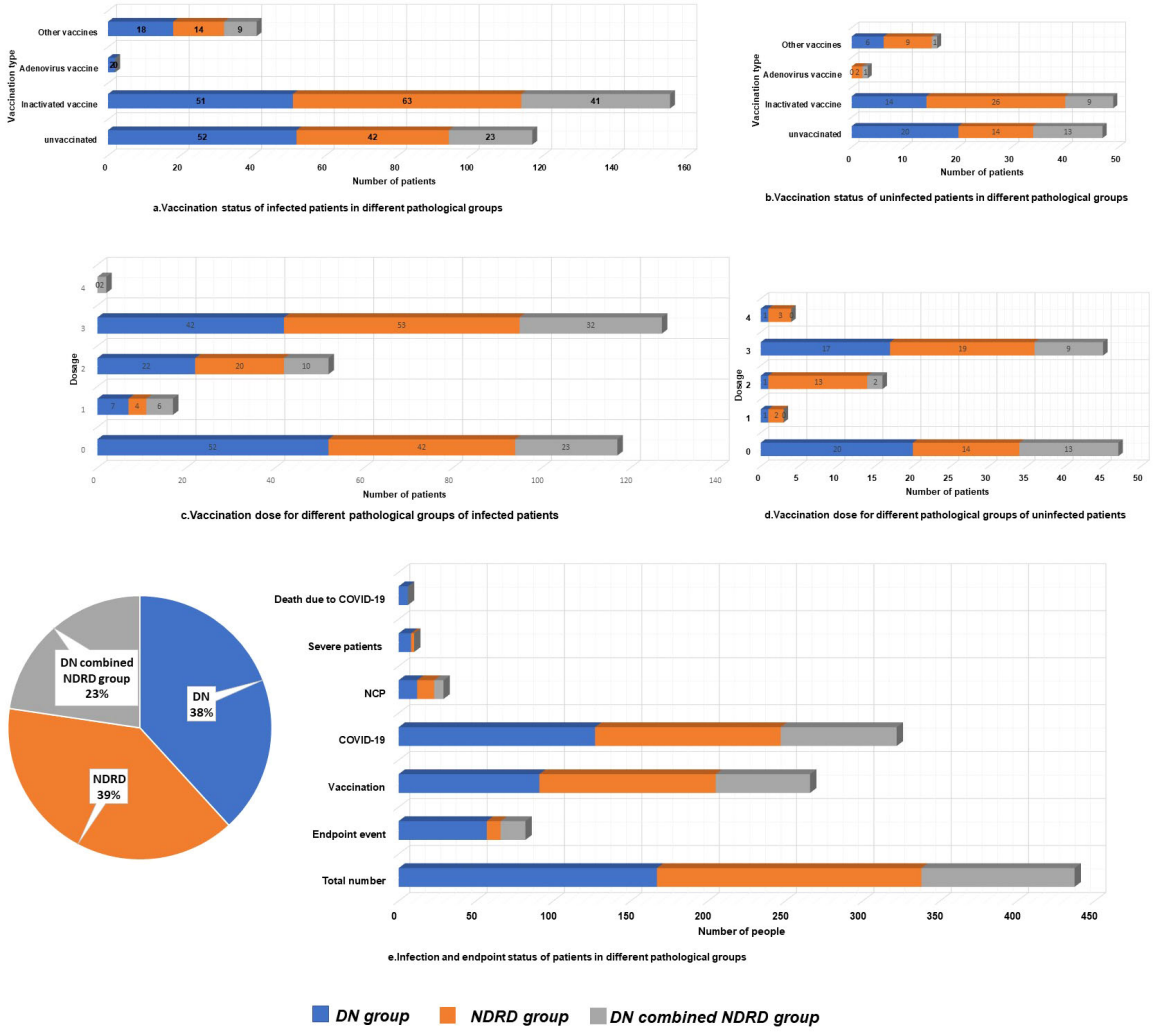
Vaccination, infection, and endpoint status of patients in different pathological groups.

Patients grouped by SARS-CoV-2 infection status exhibited comparable characteristics (**Table 1**). Clinical and renal pathological features, along with vaccination rates and dosages, were similar across both groups (**Figures 1a–d**). Predominant renal pathologies differed—DN in the infected group (127 patients, 39.4%) and NDRD in the uninfected group (51 patients, 44.3%)—yet the subtype distribution showed no significant difference. Additionally, all-cause mortality and follow-up endpoint rates were consistent between groups.

**Table 1:** Clinical characteristics and vaccination status of 437 DKD patients with or without infection.

Parameter	Infected group	Uninfected group	P-value	Χ 2
N	322	115		
Gender (male,%)	229 (71.1%)	78 (67.8%)	0.507	0.439
Age (years)	56.0 ± 10.03	57.6 ± 9.93	0.085	
Renal puncture age (years)	52.63 ± 9.88	53.6 ± 10.20	0.304	
Medical history of diabetes (month)	115.15 ± 87.82	105.95 ± 85.99	0.339	
Diabetic retinopathy (N, %)	107 (33.5%)	34 (30.6%)	0.574	0.317
Systolic blood pressure (mmHg)	124.00 (134.00,147.00)	140.50 (128.00,155.50)	0.07	
Diastolic blood pressure (mmHg)	82.3 ± 13.00	84.0 ± 12.48	0.221	
Body Mass Index (Kg/m2)	26.60 (24.62, 29.94)	26.79 (25.30, 29.19)	0.325	
White blood cells (10^9/L)	6.73 (5.47,7.81)	7.22 (6.00,8.08)	0.345	
Red blood cell (10^12/L)	4.17 ± 0.80	3.99 ± 0.76	0.037	
Hemoglobin (g/L)	123.73 ± 25.47	121.00 ± 23.44	0.319	
Alanine transaminase (U/L)	14.90 (12.73, 18.63)	15.80 (13.58, 25.00)	0.222	
Aspartate transaminase (U/L)	13.75 (11.18, 19.65)	16.45 (11.83, 25.35)	0.746	
Total serum protein (g/L)	58.13 ± 10.32	57.36 ± 11.67	0.51	
Serum Albumin (g/L)	33.19 ± 7.82	33.12 ± 10.80	0.946	
Fasting blood glucose (mmol/L)	5.58 (4.71, 6.92)	5.92 (4.83, 7.70)	0.183	
Glycosylated hemoglobin (%)	6.45 (5.90, 7.80)	6.70 (6.10, 8.23)	0.76	
Total cholesterol (mmol/L)	5.05 (4.01, 5.99)	4.89 (3.75, 5.93)	0.755	
Triglycerides (mmol/L)	2.26 (1.59, 3.23)	1.91 (1.25, 2.37)	0.23	
Low density lipoprotein (mmol/L)	3.05 (2.33, 4.05)	3.07 (2.20, 4.16)	0.344	
High density lipoprotein (mmol/L)	0.98 (0.85, 1.24)	1.11 (0.87, 1.44)	0.671	
Glomerular filtration rate (ml/min1.73m^2)	71.55 (40.68, 95.03)	64.01 (40.92, 89.35)	0.925	
Serum creatinine (umol/L)	93.30 (77.78, 154.65)	108.85 (73.60, 142.63)	0.652	
Urea nitrogen (mmol/L)	7.02 (5.33, 10.01)	6.68 (4.97, 10.43)	0.774	
Uric acid (umol/L)	367.25 (297.08,445.03)	352.85 (317.00, 433.10)	0.008	
Serum cystatin C (mg/L)	1.24 (0.96, 1.71)	1.29 (1.06, 1.91)	0.469	
24-hour urine protein (g/24h)	2.93 (1.19, 5.29)	2.25 (1.01, 4.99)	0.56	
Endpoint rate (n,%)	59 (18.3%)	23 (20%)	0.697	0.156
All-cause mortality rate (n,%)	6 (1.9%)	0 (0%)	0.14	2.173
Pathological composition			0.409	1.786
DN (n,%)	127 (39.4%)	40 (34.8%)		
NDRD (n,%)	120 (37.3%)	51 (44.3%)		
DN combined NDRD (n,%)	75 (23.3%)	24 (20.9%)		
Vaccination rate (n,%)	198 (61.5%)	68 (59.1%)	0.656	0.198
Vaccination dose			0.754	6.149
No dose (n,%)	124 (38.50%)	47 (40.90%)		
One dose (n,%)	17 (5.30%)	3 (2.60%)		
Two doses (n,%)	52 (16.10%)	16 (13.90%)		
Three doses (n,%)	127 (39.40%)	45 (39.10%)		
Four doses (n,%)	2 (0.60%)	4 (3.50%)		

DKD, Diabetic Kidney Disease; DN, Diabetic Nephropathy; NDRD, Non-Diabetic Renal Disease.

### Vaccination and infection status among pathological groups

Patients were grouped based on renal pathological subtype, as follows: DN (167, 38.22%), NDRD (171, 39.13%), and DN combined with NDRD (99, 22.65%) (**Figure 1**). In total, 82 patients reached the endpoint of follow-up (18.80%), with significant differences in rates of reaching the renal endpoint among patients with different renal pathologies. Among those reaching the endpoint of follow-up, 57 were in the DN group (34.10%), nine were in the NDRD group (5.30%), and 16 were in the DN combined with NDRD group (16.20%). Among them, a total of six patients (1.40%) died, all of whom were in the DN group (P=0.007). Rates of vaccination for the DN, NDRD, and combined DN and NDRD groups were 54.5% (91 cases), 66.7% (114 cases), and 61.6% (61 cases), respectively (P=0.071).

The COVID-19 infection rates for the groups were 76.0% (127 cases), 70.2% (120 cases), and 75.8% (75 cases), respectively. The NCP infection rates for the three groups were 7.20% (12 cases), 6.4% (11 cases), and 6.1% (6 cases), respectively. Infection and pneumonia rates did not significantly differ among the groups. Importantly, 10 patients developed severe pneumonia, and the rate of severe pneumonia in the DN group was 4.8% (eight cases), which was significantly higher than that in the NDRD group (1.2%, two cases) and the DN combined with NDRD group (0%) (P=0.019). The vaccination rate in the DN group was lower at 54.5% compared to that of the other groups. Additionally, the DN group had the highest all-cause mortality rates (3.6%, P=0.007), with 34.10% of patients reaching the follow-up endpoint (P<0.001). No pathological subtype-based differences in infection rates, vaccine coverage rates, or dosages received were observed; however, there were significant differences in disease severity and rates of reaching the follow-up endpoint (**Table 2**, **Figure 1e**).

**Table 2:** Infection, vaccination, and prognosis of patients in different pathological groups.

Parameter	Total	DN group	NDRD group	DN combined with NDRD group	P-value	χ2
N	437	167	171	99		
COVID-19 infection rate (n,%)	322 (73.7%)	127 (76.0%)	120 (70.2%)	75 (75.8%)	0.409	1.1786
Pneumonia rate (n,%)	29 (6.63%)	12 (7.20%)	11 (6.4%)	6 (6.1%)	0.93	0.146
Severity pneumonia rate (n,%)	10 (2.3%)	8 (4.8%)	2 (1.2%)	0 (0%)	0.019	7.951
Renal endpoint rate (n,%)	82 (18.80%)	57 (34.10%)	9 (5.30%)	16 (16.20%)	<0.001	46.761
All-cause mortality rate (n,%)	6 (1.4%)	6 (3.6%)	0 (0.00%)	0 (0.00%)	0.007	9.836
Vaccination rate (n,%)	266(60.9%)	91 (54.5%)	114 (66.7%)	61 (61.6%)	0.071	5.288
Vaccination dose					0.334	9.104
No dose (n,%)	171 (39.1%)	76 (45.5%)	57 (33.3%)	38 (38.4%)		
One dose (n,%)	20 (4.6%)	8 (4.8%)	6 (3.5%)	6 (6.1%)		
Two doses (n,%)	68 (15.6%)	23 (13.8%)	33 (19.3%)	12 (12.1%)		
Three doses (n,%)	172 (39.4%)	59 (35.3%)	72 (42.1%)	41 (41.4%)		
Four doses (n,%)	6 (1.4%)	1 (0.6%)	3 (1.8%)	2 (2.0%)		

DN, Diabetic Nephropathy; NDRD, Non-Diabetic Renal Disease.

### Clinical characteristics of infected patients and factors influencing vaccination

Of 322 infected patients, 114 (35.40%) had a positive nucleic acid test, 180 (55.90%) had a positive antigen test, and 28 (8.70%) were diagnosed with COVID-19 clinically without specified criteria. The vaccinated group comprised 198 patients, whereas the unvaccinated group included 117. No significant differences were observed in renal pathology between the groups. Notably, the vaccinated group exhibited a shorter diabetes history, lower mean systolic blood pressure, and higher hemoglobin, albumin, serum iron, and estimated glomerular filtration rate (eGFR) levels compared to the unvaccinated group. The opposite was found for lower serum creatinine, cystatin C, and proteinuria levels, suggesting better overall health (**Table 3**). Binary logistic regression identified serum albumin (OR=1.075, 95% CI: 1.034–1.117) and eGFR (OR=1.016, 95% CI: 1.006–1.026) as key factors influencing vaccination status among infected patients.

**Table 3:** Clinical characteristics of 322 infected patients with or without COVID-19 vaccine.

Parameter	Vaccinated group	Unvaccinated group	P-value	χ 2
N	198	117		
Gender (male,%)	147 (74.2%)	77 (65.8%)	0.111	2.544
Age (years)	55.03 ± 9.99	57.6 ± 10.16	0.027	
Renal puncture age (years)	51.75 ± 9.81	53.62 ± 10.36	0.111	
Medical history of diabetes (month)	60.00 (17.25,117.00)	120.00 (22.50,192.00)	0.032	
Diabetic retinopathy (N, %)	58 (38.5%)	45 (29.7%)	0.113	2.513
Systolic blood pressure (mmHg)	134.00 (123.25,140.75)	143.00 (123.00,151.00)	0.017	
Diastolic blood pressure (mmHg)	81.43 ± 12.31	84.12 ± 14.07	0.078	
Body Mass Index (kg/m2)	26.33 (24.58,29.03)	26.51 (23.71,31.16)	0.892	
White blood cells (10^9/L)	7.05 (5.47,7.91)	6.75 (6.03,8.37)	0.04	
Red blood cell (10^12/L)	4.29 ± 0.76	4.00 ± 0.81	0.002	
Hemoglobin (g/L)	128.15 ± 23.40	117.23 ± 26.95	0.001	
Alanine transaminase (U/L)	14.65 (11.63,19.50)	16.10 (13.90,19.55)	0.427	
Aspartate transaminase (U/L)	13.85 (10.73,21.30)	14.60 (10.40,19.95)	0.037	
Total serum protein (g/L)	60.37 ± 9.82	54.52 ± 10.31	0.001	
Serum Albumin (g/L)	34.98 ± 7.13	30.34 ± 8.21	0.001	
Fasting blood glucose (mmol/L)	5.54 (4.67, 7.59)	5.73 (4.58, 6.89)	0.68	
Glycosylated hemoglobin (%)	6.35 (5.90, 7.80)	6.40 (5.65, 7.30)	0.144	
Total iron binding force (μmol/L)	45.92 ± 10.08	40.38 ± 10.64	0.366	
Total cholesterol (mmol/L)	5.01 (3.88, 5.79)	5.10 (4.29, 5.91)	0.001	
Triglycerides (mmol/L)	2.31 (1.49, 3.62)	2.59 (1.95, 2.96)	0.109	
Low-density lipoprotein (mmol/L)	2.75 (2.04, 3.72)	3.09 (2.50, 4.11)	0.002	
High-density lipoprotein (mmol/L)	0.98 (0.84,1.31)	1.03 (0.90,1.19)	0.08	
Serum iron (umol/L)	14.75 ± 6.32	12.91 ± 5.08	0.017	
Glomerular filtration rate (ml/min1.73m^2)	86.17 (66.23,98.75)	38.71 (25.01,89.54)	0.001	
Serum creatinine (umol/L)	85.30 (73.95,107.53)	130.20 (76.75, 252.60)	0.001	
Urea nitrogen (mmol/L)	6.71 (4.48,7.91)	8.72 (5.52,11.88)	0.001	
Uric acid (umol/L)	377.95 (279.00,446.00)	369.80 (303.25,455.60)	0.441	
Serum cystatin C (mg/L)	1.07 (0.92, 1.50)	1.59 (1.02,2.91)	0.001	
24-hour urine protein (g/24h)	2.19 (1.14,4.28)	4.44 (1.58, 6.97)	0.001	
Severity rate (n,%)	2 (1.0%)	4 (3.4%)	0.131	2.284
Pneumonia rate (n,%)	13 (6.6%)	16 (13.7%)	0.035	4.447
Endpoint rate (n,%)	18 (9.10%)	37 (31.60%)	0.001	21.91
All-cause mortality rate (n,%)	0	2 (1.70%)	0.065	3.406
Pathological composition			0.279	2.556
DN (n,%)	71 (35.90%)	52 (44.40%)		
NDRD (n,%)	77 (38.90%)	42 (35.90%)		
DN combined NDRD (n,%)	50 (25.30%)	23 (19.70%)		

DN, Diabetic Nephropathy; NDRD, Non-Diabetic Renal Disease.

**Figure 2:**
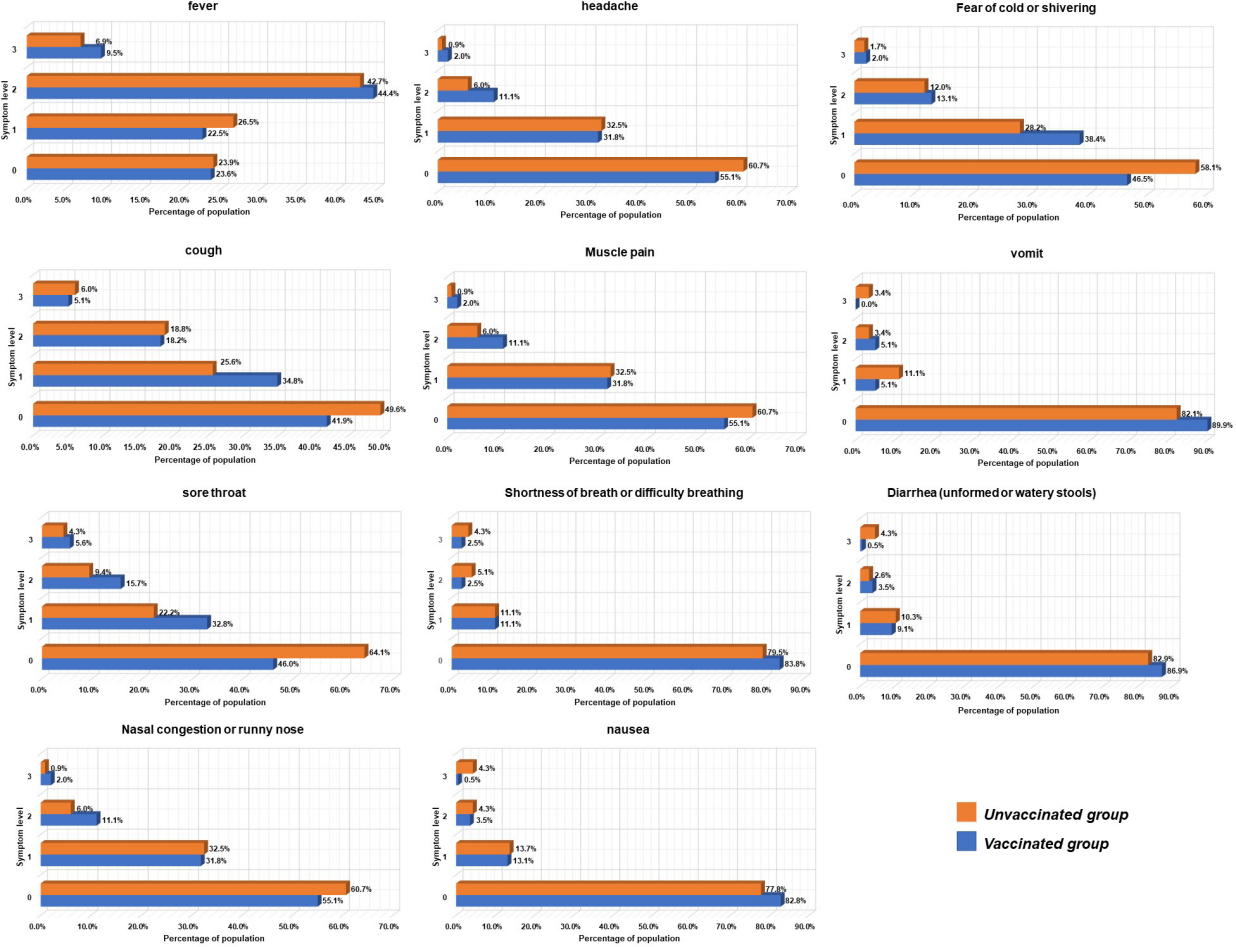
Symptom and severity scores in vaccinated and unvaccinated Groups.

The analysis of COVID-19 symptoms in unvaccinated and vaccinated patients is depicted in **Figure 2**. No significant differences were observed in the primary symptoms among infected individuals, which predominantly included fever (72.59%), cough (52.41%), chills (46.69%), sore throat (44.88%), and muscle pain (44.58%). The average body temperature was 38.5°C, and the average fever duration was 2 days in infected individuals. Vaccinated patients experienced a shorter duration of upper respiratory symptoms compared to non-vaccinated patients (5.0 vs. 7.0 days, respectively).

The NCP (6.6% vs. 13.7%), severity (1.0% vs. 3.4%), and endpoint (9.10% vs. 31.60%) rates in the vaccinated group were significantly lower than those in the unvaccinated group, with notable differences. Notably, 83.84% of vaccinated patients received treatment at home, while 28.21% of unvaccinated patients relied on community and hospital treatment. Usage rates of antiviral drugs (1.01% vs. 7.69%), antibiotics (17.17% vs. 35.04%), and respiratory support and oxygen therapy (2.53% vs. 11.11%) in the vaccinated group were significantly lower than in the unvaccinated group (P < 0.001).

## Discussion

This study elucidates the epidemiological traits and COVID-19 vaccination status of DKD patients. Our analysis showed that patients with DKD experienced a high SARS-CoV-2 infection rate (73.68%) but a low NCP rate (6.63%), with severe pneumonia or COVID-19-related deaths constituting only 2.29%. Among renal pathological subtypes, the DN group registered the lowest vaccination uptake (54.5%), highest all-cause mortality (3.6%), and endpoint of follow-up rates (34.10%). Vaccinated individuals typically exhibited improved kidney function and nutrition. The study further identified that vaccination does not significantly alter infection symptoms but effectively reduces the incidence and severity of COVID-19 pneumonia.

According to WHO COVID-19 dashboard data, at the time that the survey was conducted, the global SARS-CoV-2 infection rate is 10.01%, and the COVID-19 mortality rate is 0.91%. In contrast, China’s SARS-CoV-2 infection rate is 7.04%, and the COVID-19 mortality rate is 0.12% (W.H.O, 2023). Between December 31, 2021, and March 16, 2022, 96.26 million people in Hong Kong (12.96%) had confirmed SARS-CoV-2 infection, with severe disease occurring in 0.9% and deaths occurring in 0.7% of cases (McMenamin et al., 2022). The risk of COVID-19-related death was very low among the population of Qatar during the COVID-19 pandemic, with only 0.13 deaths among every 1,000 individuals per year (AlNuaimi et al., 2023). In a December 21, 2022, teleconference, China’s National Health Commission reported 248 million viral infections from December 1–20, 2022, with a national infection rate of 17.56% and that of some provinces above 50%. Our DKD cohort showed a higher infection rate than did the general population, yet a lower pneumonia rate (6.63%) and a higher mortality rate (1.37%). Diabetes impairs immune function, increasing COVID-19 susceptibility. Both diabetes and CKD are risk factors for SARS-CoV-2 infection and mortality, justifying our cohort’s higher mortality rate. However, it is significantly lower than previously reported mortality rates in China in 2020 [4.3% (Wang et al., 2020), 28.27% (Zhou et al., 2020), 51.7% (Yang et al., 2020)] and for 208 countries distributed globally (5.4%).

Compared with those of patients with other kidney diseases reported domestically and internationally, pneumonia and mortality rates of our cohort of patients were relatively low. A retrospective cohort study of COVID-19 in individuals with CKD in England showed that of those with prevalent and incident CKD, 6.7% and 7.8% were infected, 1.8% and 1.7% died from COVID-19, and 8.9% and 7.0% died from all causes, respectively (Dashtban et al., 2022). The COVID-19 infection rate among a hemodialysis cohort in Turkey was 13.12%, and the mortality rate was 2.87% (Kahvecioglu et al., 2023). Infection and mortality rates of the dialysis cohort in Turkey were higher than those of our population. In another study that considered patients on hemodialysis in Tirana, Albania, the prevalence of COVID-19 infection was 30.5%, and the mortality rate was 19.2%, a value higher than that for patients with DN (Rista et al., 2023). The COVID-19 pneumonia and mortality rates in our diabetic nephropathy cohort were lower than those reported previously for CKD and dialysis patients.

Diabetes has been reported to be associated with an increased risk of death among patients with COVID-19 (Alissa et al., 2023). Diabetes mortality increased significantly in the United States during the pandemic (Yao et al., 2023). In Ontario, 24% of adults with diabetes died compared to 15% of adults without diabetes. In Denmark, 16% of adults with diabetes die in the hospital compared to 13% of those without diabetes (Bogler et al., 2023).

COVID-19 morbidity and mortality are increased via unknown mechanisms in patients with diabetes and kidney disease (Menon et al., 2020). Bornstein et al. (Bornstein et al., 2020) proposed that COVID-19 infection may reduce the expression of angiotensin-converting enzyme 2 (ACE2), causing cell damage, excessive inflammation, and respiratory failure. Acute hyperglycemia upregulates ACE2 expression in cells, enhancing viral entry, while chronic hyperglycemia downregulates it, increasing vulnerability to viral inflammation and destruction. Diabetes predisposes individuals to severe COVID-19. Moreover, infection may precipitate new glucose-related complications. Elevated ACE2 expression and cellular susceptibility to SARS-CoV-2 in diabetic environments are evident in human kidney organoids and patient cells (Menon et al., 2020; Garreta et al., 2022). In addition, neuropilin-1 seems to play an important role in mechanisms linking COVID-19 to diabetic nephropathy (Mourad et al., 2021). Pre-existing endothelial dysfunction and microvascular disease in diabetes exacerbate vascular insults associated with COVID-19, leading to COVID-19 of increased severity (Basra et al., 2022).

Our study identified fever (72.59%), cough (52.41%), chills (46.69%), sore throat (44.88%), and muscle pain (44.58%) as the most prevalent symptoms in infected patients. Less common were shortness of breath, nausea, vomiting, and diarrhea. The mean age of patients was 56 years, with 23.6% being older adults (>60 years). These results align with global studies but show slight variations. A similar retrospective observational study in Pakistan reported fever (87.9%), cough (79.1%), and shortness of breath (55.1%) as primary symptoms upon hospital admission in patients with COVID-19 (Akhtar et al., 2021). In another study conducted at a screening clinic during the early outbreak period, common symptoms were fever (72%), cough (59.5%), and shortness of breath (57%). Our study found that 9.32% of patients were asymptomatic, possibly due to vaccine development and a decrease in viral strain pathogenicity.

Vaccination is a priority among patients with CKD, and the management of long-term conditions is important during and after the pandemic (Dashtban et al., 2022). A population-based study in Israel considered real-world data to evaluate the effectiveness of Paxlovid, revealing that 75.1% of patients had an adequate COVID-19 vaccination status (Najjar-Debbiny et al., 2023). At the time that the survey was conducted, China has provided 3.49 billion doses of the COVID-19 vaccine to a total of 1.3 billion people, with 1.27 billion people receiving a complete primary series of a COVID-19 vaccine. Nationally, the percentage of those receiving the first vaccine dose and that of those undergoing a complete vaccination program have reached 93.0% and 90.6%, respectively (Chinese CDC, 2023). Our study revealed a 59.3% vaccination rate in the DKD population and 54.5% in patients diagnosed with DN. Vaccinated individuals typically exhibit enhanced blood pressure, renal function, and nutritional status, unlike the unvaccinated. However, vaccine status shows a weak correlation with general clinical manifestations. Further, vaccine status is influenced by multiple factors, including willingness, infrastructure and health systems, legal and political influences (Terry et al., 2022), sociodemographic indices, and risk perception (Bayati et al., 2022).

Vaccination not only protects vulnerable individuals from SARS-CoV-2 infection but may also diminish the disease severity risk of COVID-19-related death (Zhang et al., 2023). A case-control analysis showed that unvaccinated patients accounted for 91.0% of COVID-19-related deaths, indicating that the COVID-19 vaccine may significantly reduce mortality and mechanical ventilation rates (Tenforde et al., 2021). In a study conducted in the Kingdom of Saudi Arabia, only 2.8% of mortality occurred in patients vaccinated against the SARS-CoV-2 virus (Alissa et al., 2023). Vaccination, while not significantly impacting infection rates, notably decreased pneumonia and mortality rates and lessened the need for antiviral drugs and respiratory support in vaccinated patients compared to unvaccinated ones. This suggests vaccination’s effectiveness in preventing pneumonia and reducing mortality. The similar infection rates between vaccinated and unvaccinated groups might stem from lifestyle factors, such as more cautious behavior (social distancing, mask-wearing, hand sanitization) among apprehensive patients with type 2 diabetes mellitus during the COVID-19 pandemic (Kaplan Serin and Bülbüloğlu, 2023).

Patients with DKD are closely associated with severe COVID-19, and those with varying renal pathologies exhibit different prognoses and risks of severe disease. Previous studies have not addressed COVID-19 infection in patients with DKD based on a clear pathological diagnosis. For the first time globally, we have elucidated the infection rates, vaccination statuses, and clinical profiles of patients with DKD, rooted in definitive pathological diagnoses. This provides crucial insights into how COVID-19 impacts this vulnerable group and underscores the importance of vaccination in mitigating infection severity.

The limitation of this study lies in deriving data solely from follow-up questionnaires, hence omitting neocoronavirus antibody titer, immune inflammation, and other infection markers, as well as lung CT results. The severity of COVID-19 may be associated with IgG antibody levels (Garcia-Beltran et al., 2021). Patients with severe COVID-19 exhibit elevated IgG antibody concentrations during the recovery phase (Yuan et al., 2021; Chiu et al., 2022). In these patients, the initial antibody response is delayed; however, it is notably enhanced in the middle and later stages. Specifically, IgG antibody levels in severe cases increase significantly in these stages, being 1.5 times higher than in patients exhibiting mild to moderate symptoms (Li et al., 2020). This phenomenon likely occurs because more severe infections elicit stronger immune responses. Notably, the presence of COVID-19 antibodies can serve as an indicator of disease severity and survival rates (Garcia-Beltran et al., 2021; Joyner et al., 2021).

With the global epidemic stabilizing and societal activities resuming, it is crucial to recognize that COVID-19 may hasten nephropathy progression in DN patients with compromised renal function and weakened immunity. Emphasis should be placed on infection prevention and implementing comprehensive diabetes management for these patients (Bornstein et al., 2020).

In conclusion, patients with diabetic nephropathy are vulnerable to COVID-19. Vaccination significantly reduces SARS-CoV-2 infection and lessens COVID-19 severity in DKD patients; thus, enhancing vaccination efforts and protective measures is crucial.

## Data Availability

The datasets generated and analyzed during the present study are available from the corresponding author on reasonable request.
