# Supplementary figures and images for: COVID-19 epidemic investigation study of a follow-up cohort of patients with diabetic kidney disease
Source: Front Cell Infect Microbiol. 2024 Aug 20;14:1388260. doi: 10.3389/fcimb.2024.1388260 (PMC11368908; doi:10.3389/fcimb.2024.1388260)

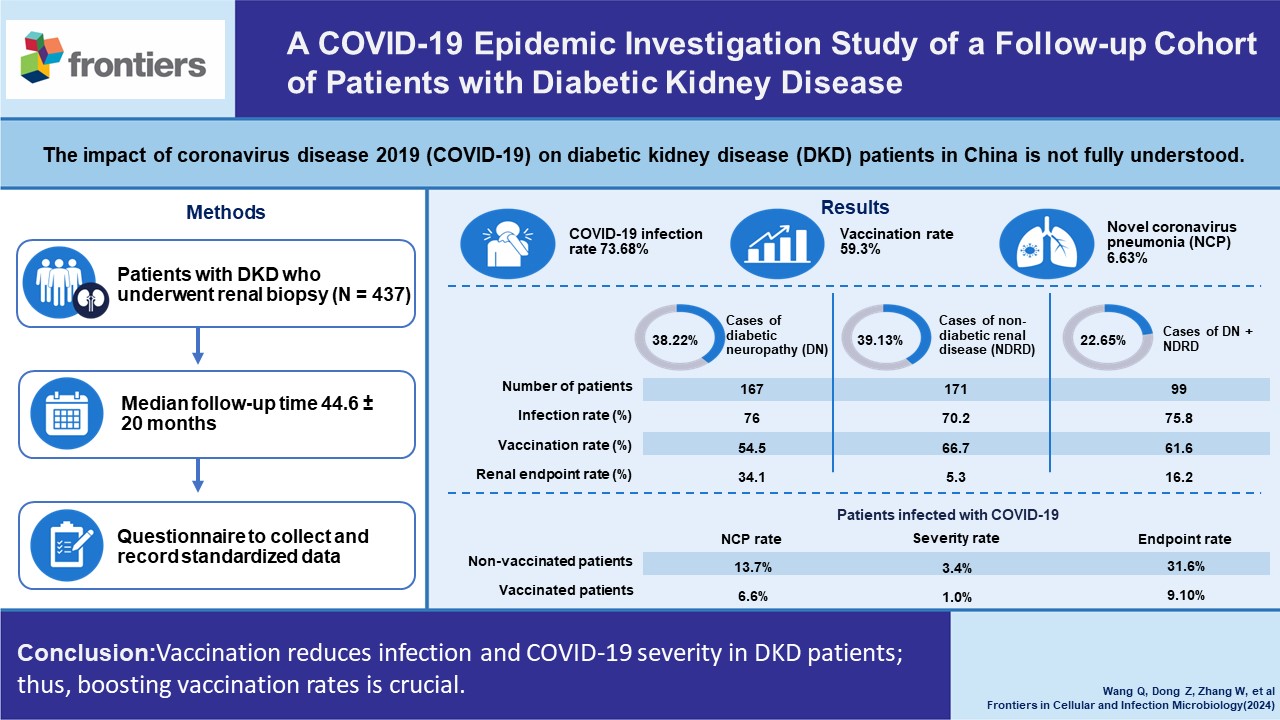

Supplement: Supplementary file 1 [file Image1.jpeg]
