# Supplementary material for: COVID-19 epidemic investigation study of a follow-up cohort of patients with diabetic kidney disease
Source: Front Cell Infect Microbiol. 2024 Aug 20;14:1388260. doi: 10.3389/fcimb.2024.1388260 (PMC11368908; doi:10.3389/fcimb.2024.1388260)
Supplement: Supplementary file 2 [file Table1.docx]

Supplementary Material

**Questionnaire on COVID-19 Infection and Vaccination of Diabetic Kidney Disease**

| Vaccination status | |
| --- | --- |
| Vaccination frequency | □No dose □ 1 dose □ 2 doses □ 3 doses □ 4 doses |
| Vaccination types | □ Inactivated vaccine □ Adenovirus vaccine □ Other |
| Diagnosis of COVID-19 infection | |
| Whether infected | □ Infected=1 □ Uninfected=0 |
| Diagnosis method | □Nasal swab nucleic acid positive; □Pharyngeal swab nucleic acid positive; □Nasal swab antigen positive; □Pharyngeal swab antigen positive; □Not tested for nucleic acid or antigen, but confirmed by family members or with family aggregation fever; □ Other |
| Time of diagnosis of COVID-19 | specific date |
| Pulmonary CT examination results | □ Not inspected; □ No obvious inflammatory changes were found during the examination; □ Diagnosed as pneumonia through examination |
| Inflammation situation | White blood cells, neutrophils, IL-6, CRP, etc |
| Clinical classification  （Doctor's diagnosis） | □ Mild □ Medium □ Severe □Critically Severe |
| Infection Symptoms and Severity Score (The most severe level) | |
| 1. Heat generation | □ None=0; Mild=1 body temperature 37.3 ℃ -38 ℃; □ Moderate=2 body temperature 38.1 ℃ -39 ℃; □ Severe=3 body temperature exceeding 39 ℃  Duration of heating days, maximum temperature ℃ |
| 2. Cough | □ None=0 □ Mild=1 □ Moderate=2 □ Severe=3 |
| 3. sore throat | □ None=0 □ Mild=1 □ Moderate=2 □ Severe=3 |
| 4. Stuck or runny nose | □ None=0 □ Mild=1 □ Moderate=2 □ Severe=3 |
| 5. Headache | □ None=0 □ Mild=1 □ Moderate=2 □ Severe=3 |
| 6. Muscle pain | □ None=0 □ Mild=1 □ Moderate=2 □ Severe=3 |
| 7. Shortness of breath or difficulty breathing | □ None=0 □ Mild=1 □ Moderate=2 □ Severe=3  Respiratory rate>30 beats per minute □ Yes □ No |
| 8. Nausea | □ None=0 □ Mild=1 □ Moderate=2 □ Severe=3 |
| 9. Fear of cold or shivering | □ None=0 □ Mild=1 □ Moderate=2 □ Severe=3 |
| 10. Vomiting | □ No=0 □ 1-2 times=1 □ 3-4 times=2 □ 5 times and above=3 |
| 11. Diarrhea | □ No=0 □ 1-2 times=1 □ 3-4 times=2 □ 5 times and above=3 |
| 12. Other | Specific symptoms  □ None=0 □ Mild=1 □ Moderate=2 □ Severe=3 |
| Duration of upper respiratory symptoms | Day/days |
| Recovery and prognosis | □ No above symptoms; □ The above symptoms have not fully recovered; □ Further aggravation; □ Other remaining situations; □ Death |
| Treatment situation | |
| Treatment methods | □ Self-treatment at home; □Outpatient treatment; □Intravenous medication treatment in community or grassroots clinics; □Hospitalization treatment in medical institutions at or above the second level; □ICU treatment |
| Used antiviral drugs | □Yes; □No; If so, please fill in the medication information |
| Used antibacterial drugs | □Yes; □No; If so, please fill in the medication information |
| Used non-steroidal anti-inflammatory drugs | □Yes; □No; If so, please fill in the medication information |
| Used glucocorticoids | □Yes; □No; If so, please fill in the medication information |
| oxygen treatment | □Yes; □No |
| Respiratory support therapy, such as mechanical ventilation | □Yes; □No |
| Other treatments |  |

**NOTE：**

The confirmation of the severity of COVID-19 is mainly based on the Diagnosis and Treatment Plan for novel coronavirus Infection (Tenth Edition). Based on a comprehensive analysis of epidemiological history, clinical manifestations, laboratory tests, and other factors, clinical diagnosis and classification (severity) assessments are made. The specific clinical classification diagnostic criteria in the guidelines are as follows:

(A) Mild: The principal symptoms of respiratory tract infection include dry throat, sore throat, cough, and fever.

(B) Moderate: Persistent high fever exceeding 3 days or persistent symptoms such as cough and shortness of breath, yet respiratory rate (RR) remains below 30 breaths/min, and oxygen saturation exceeds 93% at rest. Imaging displays the characteristic features of COVID-19 pneumonia.

(C) Severe: Presence of any of the criteria below, with symptoms not attributable to any condition other than COVID-19: a. Shortness of breath with RR ≥ 30 breaths/min; b. Oxygen saturation ≤ 93% while breathing room air at rest; c. Arterial partial pressure of oxygen (PaO2)/Fraction of inspired oxygen (FiO2) ≤ 300 mmHg; d. Progressive worsening of clinical symptoms, with lung imaging showing significant lesion progression exceeding 50% within 24 to 48 hours.

(D) Critical severe: Fulfillment of any of the following conditions: a. Occurrence of respiratory failure necessitating mechanical ventilation; b. Development of shock; c. Concomitant organ failure requiring ICU monitoring and treatment.
